# A Targeted Mass Spectrometry Approach to Identify Peripheral Changes in Metabolic Pathways of Patients with Alzheimer’s Disease

**DOI:** 10.3390/ijms24119736

**Published:** 2023-06-04

**Authors:** Pierluigi Reveglia, Carmela Paolillo, Antonella Angiolillo, Gabriella Ferretti, Ruggero Angelico, Rossana Sirabella, Gaetano Corso, Carmela Matrone, Alfonso Di Costanzo

**Affiliations:** 1 Department of Clinical and Experimental Medicine, University of Foggia, 71122 Foggia, Italy; pierluigi.reveglia@unifg.it (P.R.); carmela.paolillo@unifg.it (C.P.); gaetano.corso@unifg.it (G.C.); 2 Centre for Research and Training in Medicine of Aging, Department of Medicine and Health Sciences “Vincenzo Tiberio”, University of Molise, 86100 Campobasso, Italy; alfonso.dicostanzo@unimol.it; 3 Division of Pharmacology, Department of Neuroscience, School of Medicine, University of Naples Federico II, 80131 Naples, Italy; gabriella.ferretti@unina.it (G.F.); sirabell@unina.it (R.S.); carmela.matrone@unina.it (C.M.); 4 Department of Agriculture, Environmental and Food Sciences, University of Molise, 86100 Campobasso, Italy; angelico@unimol.it

**Keywords:** Alzheimer’s disease, metabolomic profile, lipidomic profile, targeted mass spectrometry

## Abstract

Alzheimer’s disease (AD), a neurodegenerative disorder, is the most common cause of dementia in the elderly population. Since its original description, there has been intense debate regarding the factors that trigger its pathology. It is becoming apparent that AD is more than a brain disease and harms the whole-body metabolism. We analyzed 630 polar and apolar metabolites in the blood of 20 patients with AD and 20 healthy individuals, to determine whether the composition of plasma metabolites could offer additional indicators to evaluate any alterations in the metabolic pathways related to the illness. Multivariate statistical analysis showed that there were at least 25 significantly dysregulated metabolites in patients with AD compared with the controls. Two membrane lipid components, glycerophospholipids and ceramide, were upregulated, whereas glutamic acid, other phospholipids, and sphingolipids were downregulated. The data were analyzed using metabolite set enrichment analysis and pathway analysis using the KEGG library. The results showed that at least five pathways involved in the metabolism of polar compounds were dysregulated in patients with AD. Conversely, the lipid pathways did not show significant alterations. These results support the possibility of using metabolome analysis to understand alterations in the metabolic pathways related to AD pathophysiology.

## 1. Introduction

Alzheimer’s disease (AD) has been known, for more than 100 years, as a brain disease, characterized by the combined presence of extracellular amyloid plaques consisting of amyloid-β (Aβ) peptides and intraneuronal neurofibrillary tangles, both densely packed to form highly insoluble structures [1]. Over the last decade, it has been noted that other conditions are usually associated with AD, such as cardiovascular disease and metabolic syndrome [2,3]. Indeed, AD usually includes various comorbidities, such as obesity, diabetes, dyslipidemia, and hypertension, collectively merging with metabolic syndrome [4,5,6,7]. In addition, the high Aβ insolubility of senile plaques and meningovascular Aβ deposits and the fact that Aβ is derived from the transmembrane cleavage of an integral membrane protein, the amyloid precursor protein (APP), suggests that membrane composition may control APP localization and trafficking in neurons, thus predisposing APP to undergo β-or α secretase-mediated APP processing [8,9].

Recent progress in targeted liquid chromatography coupled with mass spectrometry (LC-MS), has facilitated the dynamic evaluation of thousands of metabolites, reflecting complex networks and pathways of downstream changes at the genomic, transcriptomic, and proteome levels [10].

Here, we examined the plasma metabolome composition using a validated metabolomics procedure in patients with AD and healthy controls to assess the disease-related alterations in the metabolic pathways. The ultimate goal was to strengthen our understanding of the role of specific metabolites that might cause further disorders in AD, and to design common strategies to prevent or control these disorders during disease progression.

## 2. Results

### 2.1. Demographics of Participants

The present study investigated the plasma samples from 20 control subjects and 20 patients with AD. The epidemiological characteristics of the enrolled patients are presented in Table 1.

The AD patients did not differ in age or sex, but had significantly reduced MMSE scores compared with those of controls (*p* < 2.86 × 10^−12^). Significant differences were also observed in the educational level, which was lower in patients with AD than in controls (*p* < 0.05).

### 2.2. Partial Least Squares Discriminant Analysis (PLS-DA) and Volcano Plot for Control and AD Subjects

A well-defined platform for mass spectrometry analysis developed by the Biocrates Life Science AG service was used to analyze the metabolomic profiles of the 20 AD and 20 control subjects.

Potential alterations in the concentration of the 630 analyzed metabolites were first explored via univariate and multivariate statistical analysis, using the MetaboAnalyst 5.0 statistical analysis module.

**Figure 1:**
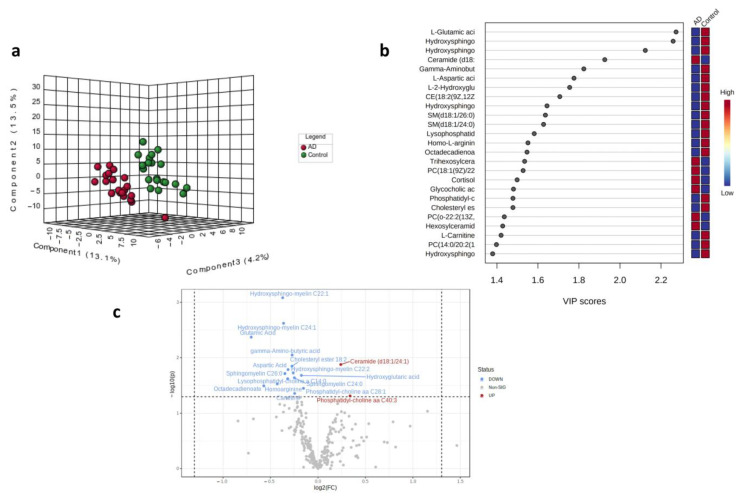
(**a**) 3D score plot of the three selected PCs for AD (red) and controls (green). The corresponding percentages of the variance (%) are reported in parentheses. (**b**) Important features identified by the PLS-DA. The boxes on the right indicate the relative concentrations of the metabolites in the AD and control. (**c**) Volcano plot with fold-change threshold (x) 1 and t-test threshold (y) of 0.05. The red circles represent features above the threshold. Note that fold changes log2 (FC) and *p* values log10 (*p*) were log transformed. On the (x) axis, the further the position from (0,0), the more significant the feature.

Figure 1 shows the results of PLS-DA. In particular, the 3D graph shows the percentage variability in the first three principal components (PC) between the patients with AD (red) and the healthy subjects (green) (Figure 1a). Patients with AD tend to have the first three components that separate them from the control subjects. The observed differences were attributable to significant differences between the concentrations of the metabolites represented in the variable importance in projection (VIP) scores (Figure 1b). Figure 1b shows that 18 of the top 25 metabolites were downregulated. Seventeen metabolites were significantly altered in the patients with AD (Figure 1c). These compounds belong to various classes of organic compounds: five sphingolipids, five amino acids, three phospholipids, two lipids, one organic acid, and one fatty acid derivative. The metabolites were represented based on fold-change values (log2 (FC)) and statistical significance (−log10 (p)). The graph shows that the main downregulated metabolites are those reported in the upper left quadrant, for example, glutamic acid, hydroxy sphingomyelin, octadecadienoate, and aspartic acid. Instead, ceramide (d18:1/24:1) appeared to be the only significantly upregulated metabolite, considering that phosphatidylcholines showed borderline variation.

Table 2 shows that most significant metabolites, with a VIP score higher than 1.7, a statistical significance (*p*-value) lower than 0.05, and a |log2FC| > 0.0: 15 were downregulated and only two were upregulated. The mean, standard deviation, standard error, range values, and 95% CI of the mean of the downregulated or/upregulated metabolites are reported in Supplementary Table S2 for comparisons between AD and controls. Pearson’s correlation analysis is reported in Supplementary Table S3. 

### 2.3. MSEA and Pathway Analysis

After the exploratory analysis, the complete data matrix with the quantified 630 metabolites was submitted to the MetaboAnalyst 5.0 modules for enrichment analysis using the MSEA algorithm, and pathway analysis.

**Figure 2:**
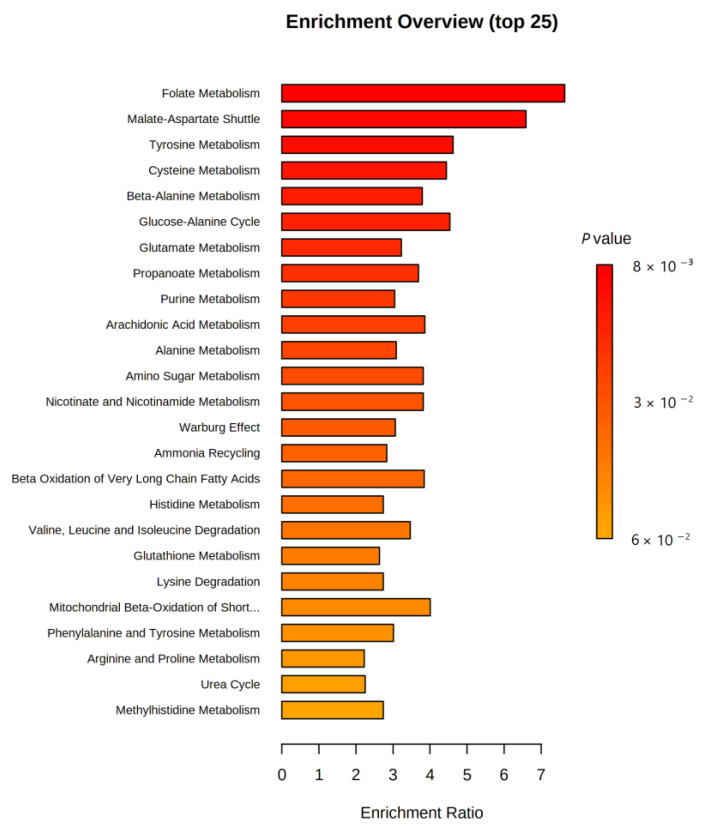
Quantitative enrichment analysis (QEA). The color of the bar refers to the *p*-value. Red bars indicate more significant enrichment.

Quantitative analysis of the metabolic pathways enriched for the 630 metabolites was not statistically significant. Nevertheless, the pathways for the biosynthesis of unsaturated fatty acids and steroid hormones were the most enriched lipids in this study. Specifically, oleic, arachidonic, docosahexaenoic, and eicosapentaenoic acids were the most enriched lipids in unsaturated fatty acid biosynthesis, whereas dehydroepiandrosterone sulfate and cortisol were the most enriched lipids in steroid hormone biosynthesis. These results are shown in Figure 2 and Table 3.

**Figure 3:**
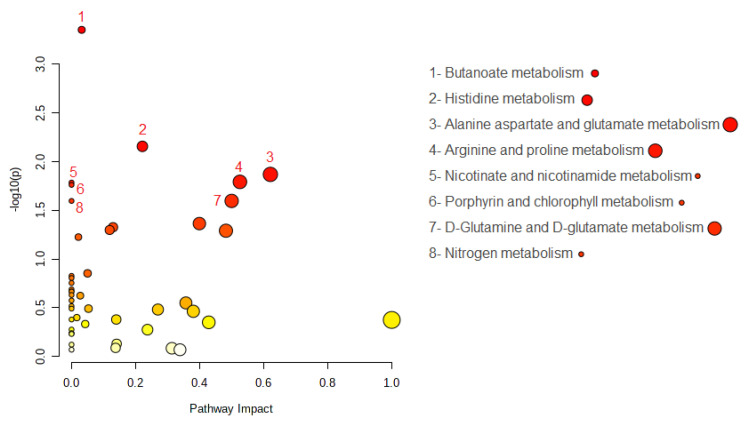
Summary of the pathway analysis. The color of the dot is the *p*-value; the redder the point, the more significant is the enrichment. The size of the spot represents the number of metabolite hits in the study.

Interestingly, eight metabolic pathways were significantly altered, and the metabolite hits for each pathway are shown in Figure 3 and Table 4. Among all the hits, L-glutamate and L-aspartate were found to be altered, which is consistent with the univariate and PLS-DA analyses reported in the previous section.

## 3. Discussion

There has been considerable speculation regarding factors besides tau protein and Aβ that may contribute to AD. Metabolic dysregulation has been reported in the brain and blood samples of patients [11,12,13,14,15]. Changes in the metabolome are detectable well before the overt clinical signs of AD and are retained as the disease progresses towards more advanced stages [16,17,18]. However, whether dysregulated metabolites play causative or modulating roles, or are by-products of AD, remains unclear. Here, we used a new and well-defined platform for mass spectrometry analysis developed by the Biocrates Life Science AG service to analyze the metabolomic profiles of 20 AD and 20 control subjects.

PLS-DA and volcano plot analysis are very useful for identifying the main components, even the latent ones, which can differentiate patients from the controls. Moreover, the PLS-DA algorithm should be regarded as just one building block in a series of steps used to develop a classification model, particularly when the study involves small sample sizes and large numbers of variables [19]. Therefore, we further investigated our data to evaluate their impact on the metabolic pathways. We, subsequently, analyzed the most significant variables, indicated by both the VIP score and volcano plot, using quantitative MSEA and pathway analysis for polar and nonpolar metabolites. The volcano plots and VIP analyses revealed an increase in the ceramide levels and a decrease in the glutamic acid levels. In this context, several other studies have reported changes in ceramide levels in the plasma of patients [20,21,22,23] or neurons in AD animal models [24,25,26]. Ceramides are precursors of sphingolipids, including sphingomyelins, gluco- and galactosyl ceramides, hexosylceramides, lactosylceramides, sphingosine, dihydrosphingosine, and dihydroceramides. The role of ceramides in AD might be consistent with their potent action as regulators of cell survival, triggering pro-apoptotic signaling pathways and leading to neurodegeneration [27]. Ceramides have also been linked to oxidative stress and mitochondrial dysfunction [28]. Alterations in the lipid raft, containing a large number of sterols (cholesterol) and sphingolipids (sphingomyelin and glycosphingolipids), have been described as being responsible for the proteolytic activities of α- or β-secretases on APP [29,30,31]. This provides a strong rationale for hypothesizing that issues with membrane restructuring may be an early cause of Aβ accumulation in the brain. Accordingly, APP trafficking into or out of lipid rafts depends on the lipid composition [15,32]. In AD neurons, owing to specific cellular signals, APP interacts with transducers or different effector proteins and accumulates in lipid raft fractions, where β- and y-secretases are mostly located and activate amyloidogenic processing [33,34,35]. Several studies have demonstrated that the dysregulation of biosynthesis, turnover, and remodeling of membrane sphingolipids promotes β peptide production and aggregation [15]. The β peptides directly disrupt the integrity of the lipid bilayer by interacting with sphingolipids [36].

In addition, alterations in the sphingolipid structure and composition affect phosphatase and tyrosine kinase activities [37,38], which are known to be altered in AD and other neurodegenerative diseases [39]. Notably, Fyn tyrosine kinase, which belongs to the Src family of protein tyrosine kinases (STKs), is an integral lipid raft protein widely expressed in the brain [39,40]. It is hyperactivated in AD and promotes either APP phosphorylation on Tyr682 [41] or Tau phosphorylation on Tyr18 [42]. In contrast, a significant reduction in plasma glutamic acid level was observed.

Glutamate is a non-essential amino acid that acts as an excitatory neurotransmitter or GABA precursor. The role of glutamate in cognitive aging and AD remains unclear, although its role in the regulation of learning and memory functions in the CNS is well recognized [43,44]. Indeed, glutamatergic synapses appear to be compromised in the AD brain, thus affecting cognition and behavior. A reduction in plasma glutamate levels was previously reported [45] and was proposed as a peripheral marker to identify patients with mild cognitive impairment (MCI) and AD [46]. A decrease in peripheral glutamate is likely paralleled by the accumulation of glutamate in the brain, with consequent neurotoxicity due to an increase in the entry of Ca^2+^ into the neurons [44]. Notably, Aβ peptides and oligomers are neurotoxic because they directly bind to glutamate receptors and activate downstream signaling, ultimately causing hyperexcitability [47,48]. Consistently, the NMDA partial antagonist, memantine, is believed to improve cognition, behavior, and global function in subjects with AD by reducing glutamate activity in the brain. Decreased glutamate levels have also been reported in the brains of patients with amnestic mild cognitive impairment (MCI), by proton magnetic resonance spectroscopy in vivo imaging (MRSI) [49]. Lin et al. reported higher L-glutamate levels in the blood of individuals with MCI and mild or moderately severe AD than in controls. In contrast, the same group showed that D-glutamate levels in the blood progressively decreased from the control to MCI, mild AD, and late-phase AD [50]. This reduction suggests that, at least in a subgroup of patients with AD, the pathogenic mechanism is characterized by an increased use of some ketogenic and/or glucogenic amino acids as an energy substrate. The carbon skeletons of ketogenic and/or glucogenic amino acids may be broken down into metabolites that enter the tricarboxylic acid (TCA) cycle to produce energy. The observation of a possible upregulation of the TCA cycle in neurodegenerative dementia, inferred from the marginal increase in several intermediates of this cycle in the serum of patients [51,52], also substantiates our hypothesis. Moreover, it is important to highlight that D-glutamate is involved in pathophysiological processes in the brains of patients with AD [45]. This finding suggests that much work needs to be conducted to elucidate the various roles of these D-amino acids in the onset and progression of AD, as recent work has clearly shown that these compounds are distinct.

Investigating the lipidome is very attractive because of its high variability over time, due to the different perturbations experienced by the human body. From an analytical perspective, the most commonly used platforms are targeted LC-MS-based techniques. High-throughput blood lipid profiling has provided insights into the crucial role of these compounds in the pathophysiology of AD. Therefore, we strongly suggest that oleic acid, arachidonic acid, docosahexaenoic acid, eicosapentaenoic acid, dehydroepiandrosterone sulfate, and cortisol should be monitored in future studies. An adequate daily intake of unsaturated fatty acids, EPA (20:5 n–3) and DHA (22:6 n–3), is officially recommended based on epidemiological results showing the beneficial role of these long-chain PUFAs n–3 in the prevention of cardiovascular and inflammatory diseases [53]. The parent fatty acid ALA (18:3 n–3), found in vegetable oils such as linseed or rapeseed oil, is used by the human body partly as an energy source and partly as a precursor of metabolites. However, the degree of endogenous conversion appears to be unreliable and limited [54]. Most human studies have shown that the conversion of high doses of ALA to EPA occurs, but to a limited extent, whereas the conversion to DHA is severely limited. Indeed, rigorous studies with radioisotopes have shown that in diets rich in saturated fats, the conversion to long-chain metabolites are approximately 6% for EPA and 3.8% for DHA. In contrast, in a diet rich in n–6 PUFA, this conversion was reduced by at least 40%. Therefore, it is reasonable to observe an n–6/n–3 PUFA ratio ≤ 2/3. Restricted conversion to DHA can be critical, as evidence has indicated that this long-chain metabolite has an autonomous function in the brain, retina, and spermatozoa, where it is the most prominent fatty acid [55].

The main limitation of the present study was the small number of participants. Another general problem affecting AD metabolomic and lipidomic studies is the lack of standardization of sampling or analytical methods, which makes it difficult to compare the results between different studies and frequently results in conflicting outcomes. The harmonization of such procedures could help scientists in this fundamental field of investigation. Although some of our findings align well with those reported in other studies [46,55,56,57], suggesting that metabolic dyshomeostasis of specific metabolites is a valuable signature of AD, there is still a significant body of literature utilizing metabolomic approaches, in which the majority of the data obtained differ depending on the patient population selected or the detection procedure applied. Therefore, to gain a greater understanding of the changes in the metabolic pathways in patients with AD, there is a need to increase the number of studies on this topic, bringing all the information together and defining common altered pathways, which might become AD signatures.

## 4. Materials and Methods

### 4.1. Participants

The subjects were recruited from the Centre for Research and Training in Medicine of Aging of the University of Molise. All subjects underwent a detailed evaluation including a neurological examination, neuropsychological tests, and routine blood tests. Patients with AD met the National Institute on Aging and the Alzheimer’s Association (NIA-AA) diagnostic criteria for “probable AD, with a documented decline” [58]. They had a Mini-Mental State Examination (MMSE) score of <24 and a Clinical Dementia Rating (CDR) of >0.5. The patient underwent brain imaging to rule out other structural causes of dementia. Subjects were classified as controls when no subjective cognitive complaint was detectable, as well as when neuropsychological tests showed normal cognitive performance with respect to age, sex, and education. The epidemiological factors of all the participants are shown in Table 1.

### 4.2. Blood Sample Collection

Blood samples were collected between 8:00 and 8:30 a.m. after a 12 h overnight fast, transferred into vacutainer tubes (Becton Dickinson, Milan, Italy), centrifuged at 2000 rpm for 15 min, and stored at −80 °C until they were analyzed.

### 4.3. Sample Analysis by MxP Quant 500 Assay by Biocrates Life Sciences

All analyses were performed by the Biocrates service using the MxP Quant 500 kit (Biocrates Life Sciences AG, Innsbruck, Austria), including the 630 metabolites.

The kit includes reagents, analytical standards, internal standards, quality control (QC) samples containing expected levels of endogenous analytes, and sample extraction plates.

Briefly, 10 μL of each plasma sample from the patients and controls were extracted in duplicates along the kit QCs and kit calibrator material by using the 96 well plate provided with the kit for sample preparation and quantitative analysis of the metabolite profiles. This device consists of inserts that have been impregnated with internal standards and a predefined sample amount was added to the inserts. After a drying step of 30 min using nitrogen, the derivatization step was performed using a 5% phenyl isothiocyanate (PITC) solution for 1 hour. After another drying step, 300 μL of 5 mM methanolic ammonium acetate was added as the extraction solvent, and the plate was shaken for 30 min, the contents were filtered into a lower sandwich plate by centrifugation at 200× *g* for 2 min. The sample extracts were diluted for subsequent analysis by mass spectrometry (MS), as specified in the kit user manual.

One extract was analyzed to identify the polar metabolites, including amino acids and related compounds, biogenic amines, fatty acids, bile acids, carbohydrates and related derivatives, hormones, vitamins, indoles and derivatives, nucleobases and their derivatives, using liquid chromatography (LC) coupled with triple quadrupole mass spectrometry (QQQ MS). The other apolar extract was analyzed for lipids including acylcarnitines, phosphatidylcholines (PC, including lyso-PC), sphingomyelins, cholesteryl esters, using flow injection analysis and QQQ MS detection. The concentrations were calculated using appropriate mass spectrometry software (Sciex-Analyst 1.7.2) and a quality control process was conducted with Biocrates MetIDQ^TM^ software. Metabolite concentrations, in µM, were provided by Biocrates Life Sciences as an Excel file (Table S1).

Further details on the analytical protocol used for the metabolite profiling can be found in patents EP1897014B1 (https://patentimages.storage.googleapis.com/ee/25/76/af35210654bdc8/EP1897014B1.pdf) and EP1875401B1 (https://patentimages.storage.googleapis.com/78/a1/77/4677d9befb2d3e/EP1875401B1.pdf).

### 4.4. Data Analysis

Multivariate statistical analysis, metabolite set enrichment analysis (MSEA), and pathway analysis were performed using the freely available online tool MetaboAnalyst 5.0 (www.metaboanalyst.ca) [59]. The volcano plot describes the up- and down regulation of metabolites by using the fold-change (FC) as a ratio between the AD and control subjects, using a criterion of |log2FC| > 0.0. The significant level was selected as the *p* value threshold < 0.05 transformed as follows: |−log10(p)|. For PLS-DA analysis, the data were normalized using the median, square root transformation, and auto scaled. Only features with VIP > 1.7 were selected. The VIP scores represent the variable’s importance in the PLS-DA model, they summarize the contribution a variable makes to the model in the classification of the two groups: control and AD. Both metabolite set enrichment analysis (MSEA) and pathway analysis were performed using the two modules available in MetaboAnalyst 5.0. The MSEA is used to identify biologically meaningful patterns that are significantly enriched in quantitative metabolomic data. In conventional approaches, metabolites are evaluated individually for their significance under study conditions. Compounds that passed certain significance levels were then combined to determine if any meaningful patterns could be discerned. In contrast, the MSEA directly investigates a set of functionally related metabolites without preselecting compounds based on an arbitrary cut-off threshold. It has the potential to identify subtle but consistent changes among a group of related compounds that may go undetected using conventional approaches. The algorithm selected for the MSEA analysis was quantitative enrichment analysis (QEA). The pathway analysis module combines powerful pathway enrichment analysis results with pathway topology analysis to identify the most relevant pathways involved in the conditions under study. Pathway enrichment analysis uses compound concentration values compared to the compound lists used in the over-representation analysis. The KEGG library was selected for pathway analysis. The significance levels were selected at 0.05 for the *p*-value and 0.15 for the FDR for both the MSEA and pathway analysis.

## 5. Conclusions

The targeted mass spectrometry used in this study showed that approximately 25 metabolites were dysregulated in patients with AD compared with the controls. Glycerophospholipids and ceramides were upregulated, while glutamic acid, other phospho-lipids, and sphingolipids were downregulated. Patients with AD also showed dysregulation of at least five pathways involved in the metabolism of polar compounds. To our knowledge, this is the first time that a kit including 630 metabolites was used in the blood of patients with AD. The obtained results could be of help in understanding the metabolic pathways involved in the onset and progression of AD.

Despite some limitations related to the small sample size, this study provides an interesting picture on the use of dysregulations in the lipidomic profile as blood signatures for patients with AD. This paves the way for the possibility of stratifying patients based on changes in metabolic/lipidic blood signatures, thus paving the way for the management of the disease using medications that have been previously used for other pathologies. Indeed, the identification of AD-related blood signatures may be used as an initial diagnostic criterion that can be further analyzed with other examinations, such as PET, neuroimaging, and cerebrospinal fluid analysis of Aβ peptides.

## Figures and Tables

**Table 1:** Epidemiological factors and disease status of the study participants. Healthy volunteers (controls, n = 20) and Alzheimer’s patients (AD, n = 20).

	Controls (n = 20)	AD (n = 20)	*p*-Value
**Age (mean ± SD)**	76.85 ± 7.9	80.85 ± 5.3	0.075
**Gender (n %):**			1
**Male**	10 (50%)	8 (40%)	
**Female**	10 (50%)	12 (60%)	
**Education (mean of years ± SD)**	11.9 ± 3.7	8.35 ± 3.4	0.003406 *
**MMSE score (mean ± SD)**	29.83 ± 1.2	20.95 ± 2.9	2.86 × 10^−12^ *
**GDS (mean ± SD)**	3.05 ± 2.9	5.38 ± 3.5	0.060041
**BMI (mean ± SD)**	27.08 ± 1.6	25.3 ± 3.9	0.108194
**Comorbidities (n, %):**			
Alcohol	12 (60%)	11 (55%)	0.749
Smoker	12 (60%)	6 (30%)	0–056
Dyslipidaemia	6 (30%)	3 (15%)	0.255
Diabetes	3 (15%)	3 (15%)	1
Hypertension	9 (45%)	6 (30%)	0.327
TIA/ischemia	1 (5%)	1 (5%)	1
Cardiac ischemia	0 (0%)	2 (10%)	0.167
Prior tumors	4 (20%)	2 (10%)	0.375
**Drugs:**			
Antihypertension	9 (45%)	6 (30%)	0.327
Hypoglycaemic	3 (15%)	3 (15%)	1
Hypolipidemic	6 (30%)	3 (15%)	0.255
Antiplatelet	1 (5%)	3 (15%)	0.197
Antiacid	2 (10%)	4 (20%)	0.375

MMSE: Mini-Mental State Examination; GDS: geriatric depression scale; BMI: body mass index. * Statistically significant.

**Table 2:** Down/upregulated metabolites for comparison between AD and controls.

Compound	Class	Type	*p*-Value	FC	log2(FC)	VIP
Hydroxysphingo-myelin C22:1	sphingolipid	Down	0.0008	0.7728	−0.3718	2.775
Hydroxysphingo-myelin C24:2	sphingolipid	Down	0.0024	0.7779	−0.3624	2.552
L-Glutamic acid	amino acid	Down	0.0043	0.6142	−0.7032	2.419
Gamma-Aminobutyric acid	amino acid	Down	0.0089	0.8282	−0.272	2.231
Ceramide (d18:1/24:1)	lipid	Up	0.0133	1.1814	0.2405	2.123
CE (18:2(9Z,12Z))	lipid	Down	0.0142	0.8273	−0.2735	2.103
L-Aspartic acid	amino acid	Down	0.0164	0.8039	−0.315	2.063
Hydroxysphingo-myelin C22:2	sphingolipid	Down	0.0188	0.8346	−0.2608	2.023
SM(d18:1/26:0)	sphingolipid	Down	0.0193	0.7856	−0.3482	2.015
L-2-Hydroxyglutaric acid	organic acid	Down	0.0207	0.8858	−0.175	1.993
SM (d18:1/24:0)	sphingolipid	Down	0.0229	0.8416	−0.2488	1.963
Lysophosphatidyl-choline C14:1	phospholipid	Down	0.0238	0.8017	−0.319	1.951
Homo-L-arginine	amino acid	Down	0.0296	0.7429	−0.4287	1.883
Octadecadienoate	fatty acid derivative	Down	0.0321	0.6734	−0.5705	1.856
Phosphatidyl-choline aa C28:1	phospholipid	Down	0.0355	0.8997	−0.1524	1.823
L-Carnitine	amino acid	Down	0.0438	0.8435	−0.2456	1.752
PC aa C40:3	phospholipid	Up	0.0486	1.2641	0.3381	1.716

Legend: CE = cholesteryl ester; SM = sphingomyelin; PC = phosphatidylcholine.

**Table 3:** Table summarizing the QEA results. FDR: false discovery rate control.

Pathway	Total Metabolites	Hits Metabolites	Raw *p*	FDR
Biosynthesis of unsaturated fatty acids	36	4	0.176	0.482
Steroid hormone biosynthesis	85	2	0.238	0.482
Fatty acid degradation	39	1	0.305	0.482
Primary bile acid biosynthesis	46	4	0.321	0.482
Lysine degradation	25	1	0.684	0.777
Arachidonic acid metabolism	36	1	0.777	0.777

**Table 4:** Altered metabolic pathways (raw *p* < 0. 05, FDR < 0.15) and altered metabolites in different pathways.

Pathway	Raw *p*	FDR	Total Metabolites in the Pathway	Hits Metabolites	Altered Metabolites	Type
Butanoate metabolism	0.0004	0.02	15	4-Aminobutanoate L-Glutamate	L-Glutamate	Down
Histidine metabolism	0.0070	0.13	16	L-Glutamate L-Histidine N(pi)-Methyl-L-histidine L-Aspartate	L-Glutamate L-Aspartate	Down Down
Alanine aspartate and glutamate metabolism	0.0135	0.13	28	L-Aspartate L-Asparagine L-Alanine L-Glutamate 4-Aminobutanoate L-Glutamine	4-Aminobutanoate L-Glutamate	Down Down
Arginine and proline metabolism	0.0161	0.13	38	L-Arginine 4-Aminobutanoate Putrescine Hydroxyproline L-Proline L-Glutamate L-Ornithine	4-Aminobutanoate L-Glutamate	Down Down
Nicotinate and nicotinamide metabolism	0.0164	0.13	15	L-Aspartate	L-Aspartate	Down
Porphyrin and chlorophyll metabolism	0.0173	0.13	30	Glycine L-Glutamate	L-Glutamate	Down
D-Glutamine and D-glutamate metabolism	0.0253	0.14	6	L-Glutamate L-Glutamine	L-Glutamate	Down
Nitrogen metabolism	0.0253	0.14	6	L-Glutamate L-Glutamine	L-Glutamate	Down

## Data Availability

Datasets are available upon request from authors. All the raw spectra data are stored by Biocrates Life Sciences AG.

## Supplementary Materials

The supporting information can be downloaded at: https://www.mdpi.com/article/10.3390/ijms24119736/s1.
