# Supplementary material for: A Targeted Mass Spectrometry Approach to Identify Peripheral Changes in Metabolic Pathways of Patients with Alzheimer’s Disease
Source: Int J Mol Sci. 2023 Jun 4;24(11):9736. doi: 10.3390/ijms24119736 (PMC10253805; doi:10.3390/ijms24119736)
Supplement: Supplementary file 1 [file ijms-24-09736-s001.zip › ijms-2351419-supplementary.pdf]

Supplementary Table 1: List of the analyzed metabolites (MxP Quant 500 assay by BIOCRATES Life Sciences)

Type	Names					
Acylcarnitines	Carnitine	Butenylcarnitine	Hexanoylcarnitine (Fumarylcarnitine)	Decadienoyl-carnitine	Tetradecadienoyl-carnitine	Hydroxyhexadeca-dienoylcarnitine
	Acetylcarnitine	Valerylcarnitine	Hexenoylcarnitine	Dodecanoyl-carnitine	Hydroxytetradeca-dienoylcarnitine	Octadecanoyl-carnitine
	Propionylcarnitine	Glutarylcarnitine (Hydroxyhexanoylcarnitine)	Pimelylcarnitine	Dodecanedioyl-carnitine	Hexadecanoyl-carnitine	Octadecenoyl-carnitine
	Malonylcarnitine (Hydroxybutyrylcarnitine)	Methylglutaryl-carnitine	Octanoylcarnitine	Dodecenoyl-carnitine	Hydroxyhexa-decanoylcarnitine	Hydroxyoctadec-enoylcarnitine
	Hydroxypropionyl-carnitine	Hydroxyvaleryl-carnitine (Methylmalonylcarnitine)	Nonanoylcarnitine	Tetradecanoyl-carnitine	Hexadecenoyl-carnitine	Octadecadienoyl-carnitine
	Propenylcarnitine	Tiglylcarnitine	Decanoylcarnitine	Tetradecenoyl-carnitine	Hydroxyhexadec-enoylcarnitine	
	Butyrylcarnitine	Glutaconyl-carnitine	Decenoylcarnitine	Hydroxytetradec-enoylcarnitine	Hexadecadienoyl-carnitine	
Alkaloids	Trigonelline					
Amine Oxides	Trimethylamine N-oxide					
Amino Acid Related	1-Methylhistidine	Asymmetric dimethylarginine	cis-4-Hydroxyproline	Dihydroxy-phenylalanine	Nitrotyrosine	Sarcosine
	3-Methylhistidine	alpha-Aminoadipic acid	Carnosine	Homoarginine	Ornithine	Symmetric dimethylarginine
	5-Aminovaleric acid	L-Anserine	Citrulline	Homocysteine	Phenylacetyl-glycine	trans-4-Hydroxyproline

	alpha-Amino-butyric acid	beta-Aminobutyric acid	Creatinine	Kynurenine	Phenylalanine betaine	Taurine
	Acetylornithine	Betaine	Cystine	Methionine-Sulfoxide	Proline betaine	Tryptophan betaine
Amino Acid	Alanine	Cysteine	Histidine	Methionine	Threonine	
	Arginine	Glutamine	Isoleucine	Phenylalanine	Tryptophan	
	Asparagine	Glutamic Acid	Leucine	Proline	Tyrosine	
	Aspartic Acid	Glycine	Lysine	Serine	Valine	
Bile Acids	Cholic Acid	Glycocholic acid	Glycolithocholic acid	Taurocholic acid	Taurolithocholic acid	
	Chenodeoxycholic acid	Glycochenodeoxy-cholic acid	Glycolithocholic acid sulfate	Taurochenodeoxy-cholic acid	Tauromuricholic acids	
	Deoxycholic acid	Glycodeoxycholic acid	Glycoursodeoxy-cholic acid	Taurodeoxycholic acid		
Biogenic Amines	beta-Alanine	gamma-Amino-butyric acid	Phenylethylamine	Serotonin	Spermine	
	Dopamine	Histamine	Putrescine	Spermidine		
Carbohydrates and Related	Hexose					
Carboxylic Acids	Aconitic acid	Tetradecanedioic acid	Lactic acid	Succinic acid		
	Dodecanedioic acid	Hippuric acid	Hydroxyglutaric acid			
Ceramides	(d16:1/18:0)	(d18:1/14:0)	(d18:1/20:0(OH))	(d18:1/24:1)	(d18:2/16:0)	(d18:2/23:0)
	(d16:1/20:0)	(d18:1/16:0)	(d18:1/20:0)	(d18:1/25:0)	(d18:2/18:0)	(d18:2/24:0)
	(d16:1/22:0)	(d18:1/18:0(OH))	(d18:1/22:0)	(d18:1/26:0)	(d18:2/18:1)	(d18:2/24:1)
	(d16:1/23:0)	(d18:1/18:0)	(d18:1/23:0)	(d18:1/26:1)	(d18:2/20:0)	

	(d16:1/24:0)	(d18:1/18:1)	(d18:1/24:0)	(d18:2/14:0)	(d18:2/22:0)	
Cholesteryl Esters	14:0	16:0	18:0	20:0	20:5	22:5
	14:1	16:1	18:1	20:1	22:0	22:6
	15:0	17:0	18:2	20:3	22:1	
	15:1	17:1	18:3	20:4	22:2	
Cresols	p-Cresol sulfate					
Diacylglyceride	(14:0_14:0)	(16:0_18:1)	(16:1_20:0)	(18:1_18:4)	(18:2_18:2)	(22:1_22:2)
	(14:0_18:1)	(16:0_18:2)	(17:0_17:1)	(18:1_20:0)	(18:2_18:3)	O-(14:0_18:2)
	(14:0_18:2)	(16:0_20:0)	(17:0_18:1)	(18:1_20:1)	(18:2_18:4)	O-(16:0_18:1)
	(14:0_20:0)	(16:0_20:3)	(18:0_20:0)	(18:1_20:2)	(18:2_20:0)	O-(16:0_20:4)
	(14:1_18:1)	(16:0_20:4)	(18:0_20:4)	(18:1_20:3)	(18:2_20:4)	
	(14:1_20:2)	(16:1_18:0)	(18:1_18:1)	(18:1_20:4)	(18:3_18:3)	
	(16:0_16:0)	(16:1_18:1)	(18:1_18:2)	(18:1_22:5)	(18:3_20:2)	
	(16:0_16:1)	(16:1_18:2)	(18:1_18:3)	(18:1_22:6)	(21:0_22:6)	
Dihexosyl ceramides	(d18:1/14:0)	(d18:1/20:0)	(d18:1/24:1)	(18:0/18:0(OH)	(18:0/22:0)	(18:0/26:1(OH)
	(d18:1/16:0)	(d18:1/22:0)	(d18:1/26:0)	(18:0/18:0)	(18:0/24:0)	(18:0/26:1)
	(d18:1/18:0)	(d18:1/24:0)	(d18:1/26:1)	(18:0/20:0)	(18:0/24:1)	
Fatty Acids	Arachidonic acid	Eicosapentaenoic acid	Myristic acid	Stearic acid	Octadecadienoate	Eicosadienoic acid
	Docosahexaenoic acid	Dodecanoic acid	Palmitic acid	Octadecenoic acid	Eicosenoic acid	Eicosatrienoic acid
Hexosyl ceramide	(d16:1/22:0)	(d18:1/18:0)	(d18:1/23:0)	(d18:1/26:1)	(d18:2/22:0)	
	(d16:1/24:0)	(d18:1/18:1)	(d18:1/24:0)	(d18:2/16:0)	(d18:2/23:0)	

	(d18:1/14:0)	(d18:1/20:0)	(d18:1/24:1)	(d18:2/18:0)	(d18:2/24:0)	
	(d18:1/16:0)	(d18:1/22:0)	(d18:1/26:0)	(d18:2/20:0)		
Hormones and Related	Abscisic acid	Cortisol	Cortisone	Dehydroepiandro-sterone sulfate		
Indoles and Derivatives	Indoleacetic acid	Indolepropionic acid	Indoxyl sulfate	Indole		
Lysophosphatidyl-cholines	C14:0	C17:0	C18:2	C24:0	C28:0	
	C16:0	C18:0	C20:3	C26:0	C28:1	
	C16:1	C18:1	C20:4	C26:1		
Nucleobases and Related	Hypoxanthine	Xanthine				
Phosphatidyl-cholines	aa C24:0	aa C36:0	aa C40:1	ae C30:1	ae C36:5	ae C40:6
	aa C26:0	aa C36:1	aa C40:2	ae C30:2	ae C38:0	ae C42:0
	aa C28:1	aa C36:2	aa C40:3	ae C32:1	ae C38:1	ae C42:1
	aa C30:0	aa C36:3	aa C40:4	ae C32:2	ae C38:2	ae C42:2
	aa C30:2	aa C36:4	aa C40:5	ae C34:0	ae C38:3	ae C42:3
	aa C32:0	aa C36:5	aa C40:6	ae C34:1	ae C38:4	ae C42:4
	aa C32:1	aa C36:6	aa C42:0	ae C34:2	ae C38:5	ae C42:5
	aa C32:2	aa C38:0	aa C42:1	ae C34:3	ae C38:6	ae C44:3
	aa C32:3	aa C38:1	aa C42:2	ae C36:0	ae C40:1	ae C44:4
	aa C34:1	aa C38:3	aa C42:4	ae C36:1	ae C40:2	ae C44:5
	aa C34:2	aa C38:4	aa C42:5	ae C36:2	ae C40:3	ae C44:6
	aa C34:3	aa C38:5	aa C42:6	ae C36:3	ae C40:4	
	aa C34:4	aa C38:6	ae C30:0	ae C36:4	ae C40:5	
Sphingomyelins	Hydroxysphingo-myelin C14:1	Hydroxysphingo-myelin C22:2	Sphingomyelin C16:1	Sphingomyelin C20:2	Sphingomyelin C24:1	
	Hydroxysphingo-myelin C16:1	Hydroxysphingo-myelin C24:1	Sphingomyelin C18:0	Sphingomyelin C22:3	Sphingomyelin C26:0	

	Hydroxysphingo-myelin C22:1	Sphingomyelin C16:0	Sphingomyelin C18:1	Sphingomyelin C24:0	Sphingomyelin C26:1	
Triacylglyceride	(14:0_32:2)	(16:0_38:5)	(17:2_34:3)	(18:1_36:0)	(18:3_34:0)	(20:3_36:5)
	(14:0_34:0)	(16:0_38:6)	(17:2_36:2)	(18:1_36:1)	(18:3_34:1)	(20:4_30:0)
	(14:0_34:1)	(16:0_38:7)	(17:2_36:3)	(18:1_36:2)	(18:3_34:2)	(20:4_32:0)
	(14:0_34:2)	(16:0_40:6)	(17:2_36:4)	(18:1_36:3)	(18:3_34:3)	(20:4_32:1)
	(14:0_34:3)	(16:0_40:7)	(17:2_38:5)	(18:1_36:4)	(18:3_35:2)	(20:4_32:2)
	(14:0_35:1)	(16:0_40:8)	(17:2_38:6)	(18:1_36:5)	(18:3_36:1)	(20:4_33:2)
	(14:0_35:2)	(16:1_28:0)	(17:2_38:7)	(18:1_36:6)	(18:3_36:2)	(20:4_34:0)
	(14:0_36:1)	(16:1_30:1)	(18:0_30:0)	(18:1_38:5)	(18:3_36:3)	(20:4_34:1)
	(14:0_36:2)	(16:1_32:0)	(18:0_30:1)	(18:1_38:6)	(18:3_36:4)	(20:4_34:2)
	(14:0_36:3)	(16:1_32:1)	(18:0_32:0)	(18:1_38:7)	(18:3_38:5)	(20:4_34:3)
	(14:0_36:4)	(16:1_32:2)	(18:0_32:1)	(18:2_28:0)	(18:3_38:6)	(20:4_35:3)
	(14:0_38:4)	(16:1_33:1)	(18:0_32:2)	(18:2_30:0)	(20:0_32:3)	(20:4_36:2)
	(14:0_38:5)	(16:1_34:0)	(18:0_34:2)	(18:2_30:1)	(20:0_32:4)	(20:4_36:3)
	(14:0_39:3)	(16:1_34:1)	(18:0_34:3)	(18:2_31:0)	(20:0_34:1)	(20:4_36:4)
	(16:0_28:1)	(16:1_34:2)	(18:0_36:1)	(18:2_32:0)	(20:1_24:3)	(20:4_36:5)
	(16:0_28:2)	(16:1_34:3)	(18:0_36:2)	(18:2_32:1)	(20:1_26:1)	(20:5_34:0)
	(16:0_30:2)	(16:1_36:1)	(18:0_36:3)	(18:2_32:2)	(20:1_30:1)	(20:5_34:1)
	(16:0_32:0)	(16:1_36:2)	(18:0_36:4)	(18:2_33:0)	(20:1_31:0)	(20:5_34:2)
	(16:0_32:1)	(16:1_36:3)	(18:0_36:5)	(18:2_33:1)	(20:1_32:1)	(20:5_36:2)
	(16:0_32:2)	(16:1_36:4)	(18:0_38:6)	(18:2_33:2)	(20:1_32:2)	(20:5_36:3)
	(16:0_32:3)	(16:1_36:5)	(18:0_38:7)	(18:2_34:0)	(20:1_32:3)	(22:0_32:4)
	(16:0_33:1)	(16:1_38:3)	(18:1_26:0)	(18:2_34:1)	(20:1_34:0)	(22:1_32:5)
	(16:0_33:2)	(16:1_38:4)	(18:1_28:1)	(18:2_34:2)	(20:1_34:1)	(22:2_32:4)
	(16:0_34:0)	(16:1_38:5)	(18:1_30:0)	(18:2_34:3)	(20:1_34:2)	(22:3_30:2)
	(16:0_34:1)	(17:0_32:1)	(18:1_30:1)	(18:2_34:4)	(20:1_34:3)	(22:4_32:0)
	(16:0_34:2)	(17:0_34:1)	(18:1_30:2)	(18:2_35:1)	(20:2_32:0)	(22:4_32:2)
	(16:0_34:3)	(17:0_34:2)	(18:1_31:0)	(18:2_35:2)	(20:2_32:1)	(22:4_34:2)
	(16:0_34:4)	(17:0_34:3)	(18:1_32:0)	(18:2_35:3)	(20:2_34:1)	(22:5_32:0)
	(16:0_35:1)	(17:0_36:3)	(18:1_32:1)	(18:2_36:0)	(20:2_34:2)	(22:5_32:1)
	(16:0_35:2)	(17:0_36:4)	(18:1_32:2)	(18:2_36:1)	(20:2_34:3)	(22:5_34:1)

	(16:0_35:3)	(17:1_32:1)	(18:1_32:3)	(18:2_36:2)	(20:2_34:4)	(22:5_34:2)
	(16:0_36:2)	(17:1_34:1)	(18:1_33:0)	(18:2_36:3)	(20:2_36:5)	(22:5_34:3)
	(16:0_36:3)	(17:1_34:2)	(18:1_33:1)	(18:2_36:4)	(20:3_32:0)	(22:6_32:0)
	(16:0_36:4)	(17:1_34:3)	(18:1_33:2)	(18:2_36:5)	(20:3_32:1)	(22:6_32:1)
	(16:0_36:5)	(17:1_36:3)	(18:1_33:3)	(18:2_38:4)	(20:3_32:2)	(22:6_34:1)
	(16:0_36:6)	(17:1_36:4)	(18:1_34:1)	(18:2_38:5)	(20:3_34:0)	(22:6_34:2)
	(16:0_37:3)	(17:1_36:5)	(18:1_34:2)	(18:2_38:6)	(20:3_34:1)	(22:6_34:3)
	(16:0_38:1)	(17:1_38:5)	(18:1_34:3)	(18:3_30:0)	(20:3_34:2)	
	(16:0_38:2)	(17:1_38:6)	(18:1_34:4)	(18:3_32:0)	(20:3_34:3)	
	(16:0_38:3)	(17:1_38:7)	(18:1_35:2)	(18:3_32:1)	(20:3_36:3)	
	(16:0_38:4)	(17:2_34:2)	(18:1_35:3)	(18:3_33:2)	(20:3_36:4)	
Trihexosylceramides	(d18:1/16:0)	(d18:1/18:0)	(d18:1/24:1)	(d18:1/26:1)	(d18:1/20:0)	(d18:1/22:0)
Vitamins and Cofactors	Choline					

Supplementary Table 2. The table reports mean, standard deviation, standard error, 95% confidence interval (CI), and range values (minimum and maximum values) of the downregulated or upregulated metabolites for comparison between patients with AD and controls.

	Compound	Mean	Standard Deviation	Standard Error	95% CI of the Mean		Minimum	Maximum
					Upper Limit	Lower Limit		
**Controls**	L-Carnitine	38.057	6.589	1.473	34.973	41.140	26.440	48.750
	Homo-L-arginine	2.389	0.997	0.223	1.922	2.856	1.220	4.930
	L-Aspartic acid	10.417	3.289	0.735	8.877	11.956	6.680	21.200
	L-Glutamic acid	115.233	82.584	18.466	76.583	153.883	56.400	452.000
	L-2-Hydroxyglutaric acid	7.250	1.337	0.299	6.624	7.875	5.350	10.400
	Ceramide (d18:1/24:1)	1.128	0.245	0.055	1.013	1.242	0.730	1.810
	CE (18:2(9Z,12Z))	785.494	229.788	51.382	677.950	893.038	399.950	1306.000
	Ocatadecadienoate	28.983	18.095	4.046	20.514	37.451	9.670	97.360
	Lysophosphatidyl-choline C14:1	7.417	1.962	0.439	6.498	8.335	4.020	12.170
	Phosphatidyl-choline aa C28:1	3.045	0.566	0.127	2.779	3.310	1.910	3.750
	Hydroxysphingo-myelin C22:2	7.343	2.151	0.481	6.336	8.349	3.870	10.800
	Hydroxysphingo-myelin C22:1	0.852	0.321	0.072	0.702	1.002	0.410	1.440
	Hydroxysphingo-myelin C24:2	12.836	3.998	0.894	10.965	14.706	5.420	19.400
	SM (d18:1/24:0)	0.146	0.021	0.005	0.136	0.155	0.120	0.180
	Gamma-Aminobutyric acid	0.473	0.166	0.037	0.395	0.550	0.240	0.830
	PC aa C40:3	0.096	0.033	0.007	0.080	0.112	0.050	0.170
	Phosphatidyl-choline aa C28:1	0.096	0.033	0.007	0.080	0.112	0.050	0.170

**AD**	L-Carnitine	35.592	8.580	1.918	31.577	39.607	17.200	49.840
	Homo-L-arginine	1.999	0.994	0.222	1.533	2.464	0.640	4.770
	L-Aspartic acid	9.434	2.176	0.487	8.415	10.452	6.220	14.300
	L-Glutamic acid	79.672	19.817	4.431	70.397	88.946	50.140	122.000
	L-2-Hydroxyglutaric acid	7.230	1.512	0.338	6.522	7.938	5.120	10.100
	Ceramide (d18:1/24:1)	1.494	0.355	0.079	1.327	1.660	0.950	2.190
	CE (18:2(9Z,12Z))	726.256	180.703	40.406	641.684	810.828	387.000	1041.000
	Ocatadecadienoate	22.015	11.061	2.473	16.838	27.192	0.000	39.900
	Lysophosphatidyl-choline C14:1	6.883	2.271	0.508	5.820	7.945	3.520	11.600
	Phosphatidyl-choline aa C28:1	3.071	0.682	0.153	2.751	3.390	1.680	4.350
	Hydroxysphingo-myelin C22:2	6.769	1.534	0.343	6.051	7.487	4.250	9.650
	Hydroxysphingo-myelin C22:1	0.720	0.155	0.035	0.647	0.793	0.460	1.150
	Hydroxysphingo-myelin C24:2	11.985	2.833	0.634	10.659	13.311	7.090	18.400
	SM (d18:1/24:0)	0.135	0.027	0.006	0.123	0.147	0.080	0.190
	Gamma-Aminobutyric acid	0.670	0.295	0.066	0.532	0.808	0.320	1.310
	PC aa C40:3	0.082	0.027	0.006	0.069	0.094	0.030	0.140
	Phosphatidyl-choline aa C28:1	0.082	0.027	0.006	0.069	0.094	0.030	0.140

Supplementary Table 3. Pearson's correlation matrix of downregulated or upregulated metabolites in subjects with AD.

	L-Carnitine	Homo-L-arginine	L-Aspartic acid	L-Glutamic acid	L-2-Hydroxyglutaric acid	Ceramide (d18:1/24:1)	CE (18:2(9Z.12Z))	Ocatadecadienoate	Lysophosphatidyl-choline C14:1	Phosphatidyl-choline aa C28:1	Hydroxysphingo-myelin C22:2	Hydroxysphingo-myelin C22:1	Hydroxysphingo-myelin C24:2	SM (d18:1/24:0)	Gamma-Aminobutyric acid	PC aa C40:3	Phosphatidyl-choline aa C28:1
L-Carnitine	1.00	-0.23	0.30	-0.03	-0.04	0.32	-0.15	0.20	-0.25	-0.24	0.17	0.06	-0.07	0.14	-0.16	0.26	0.26
Homo-L-arginine		1.00	.498*	0.22	.451*	0.01	0.23	-0.03	0.00	-0.05	0.00	0.38	0.29	0.33	0.04	0.24	0.24
L-Aspartic acid			1.00	.462*	.592**	.489*	0.33	0.31	0.31	0.16	0.15	0.30	.461*	.490*	0.24	0.27	0.27
L-Glutamic acid				1.00	.520*	0.22	0.15	0.07	0.19	0.20	0.15	0.09	0.20	0.33	.463*	-0.36	-0.36
L-2-Hydroxyglutaric acid					1.00	.568**	.676**	0.16	0.21	.526*	0.43	.466*	.690**	0.37	.581**	-0.24	-0.24
Ceramide (d18:1/24:1)						1.00	0.43	0.27	0.23	0.29	0.37	0.32	.533*	0.35	0.35	-0.01	-0.01
CE (18:2(9Z.12Z))							1.00	0.14	0.42	.706**	.711**	.584**	.813**	0.06	0.15	0.14	0.14
Ocatadecadienoate								1.00	0.02	-0.10	-0.15	0.01	0.02	0.44	-0.04	0.12	0.12
Lysophosphatidyl-choline C14:1									1.00	.718**	0.34	0.27	.567**	-0.01	0.26	0.22	0.22
Phosphatidyl-choline aa C28:1										1.00	.631**	0.43	.758**	-0.02	0.42	-0.07	-0.07
Hydroxysphingo-myelin C22:2											1.00	.551*	.710**	-0.18	0.07	0.15	0.15
Hydroxysphingo-												1.00	.730**	-0.01	-0.13	.496*	.496*

myelin C22:1																	
Hydroxysphingo-myelin C24:2													1.00	-0.05	0.28	0.20	0.20
SM (d18:1/24:0)														1.00	0.13	-0.03	-0.03
Gamma-Aminobutyric acid															1.00	-.675**	-.675**
PC aa C40:3																1.00	1.000**
Phosphatidyl-choline aa C28:1																	1.00

*Significant correlation P< 0.05.

**Significant correlation P< 0.01.
